# Prenatal Exposure to Alcohol

**Published:** 2000

**Keywords:** fetal alcohol syndrome, prenatal alcohol exposure, neurodevelopmental anomaly, craniofacial anomaly, diagnostic criteria, brain damage, cognitive and memory disorder, central nervous system, neurotransmission, neuroimaging

## Abstract

Maternal alcohol consumption during pregnancy can cause serious birth defects, of which fetal alcohol syndrome (FAS) is the most devastating. Recognizable by characteristic craniofacial abnormalities and growth deficiency, this condition includes severe alcohol-induced damage to the developing brain. FAS children experience deficits in intellectual functioning; difficulties in learning, memory, problem-solving, and attention; and difficulties with mental health and social interactions. An FAS diagnosis, however, fails to identify prenatal-alcohol-exposed children who lack the characteristic facial defects and growth deficiency of FAS. Nonetheless, these often undiagnosed children may still experience serious fetal alcohol effects (FAE), including alcohol-induced mental impairments (i.e., alcohol-related neurodevelopment disorder) or alcohol-related abnormalities of the skeleton and certain organ systems (i.e., alcohol-related birth defects). Neuroimaging techniques can assist researchers in identifying FAE through precise pictures of brain abnormalities in persons prenatally exposed to alcohol. By understanding the mechanisms underlying FAE and the behavioral manifestations of the resulting structural brain damage, researchers can ultimately develop effective FAS prevention strategies that identify and assist high-risk women at varying levels of pregnancy.

Fetal Alcohol Syndrome (FAS) is a set of specific birth defects caused by maternal alcohol consumption during pregnancy. Scientists first identified the syndrome in France in 1968 (Lemoine et al. 1968) and in the United States in 1973 (Jones et al. 1973). Today, FAS is considered the most common nonhereditary cause of mental retardation. Estimates of FAS prevalence vary from 0.5 to 3.0 per 1,000 live births in most populations, with much higher rates occurring in some communities (Stratton et al. 1996).

At birth, children with FAS are recognizable by their apparent growth deficiency and characteristic minor facial anomalies (i.e., craniofacial abnormalities) that tend to become less noticeable and adopt a more normal appearance as the child matures. Less evident at birth—but far more devastating to FAS children and their families—are the lifelong effects of alcohol-induced damage to the developing brain. In addition to deficits in general intellectual functioning, persons with FAS often demonstrate difficulties with learning, memory, problem-solving, and attention as well as difficulties with mental health and social interactions.

However, the diagnosis of FAS identifies only a relatively small proportion of children affected by alcohol exposure before birth. Many children with significant prenatal alcohol exposure lack the characteristic facial defects and growth deficiency of FAS but still have serious alcohol-induced mental impairments. This condition is referred to as “alcohol-related neurodevelopmental disorder” (ARND). In addition, some prenatally exposed children without FAS facial features exhibit other alcohol-related physical abnormalities of the skeleton and certain organ systems; these anomalies are referred to as alcohol-related birth defects (ARBD).

## Diagnosing the Effects of Prenatal Alcohol Exposure

Researchers first outlined the diagnostic criteria for FAS in 1973 (Jones et al. 1973). Although the terms used to describe the condition have changed over the years, the diagnostic criteria—growth deficiency, dysfunction of the central nervous system, and characteristic facial defects—have remained essentially the same. Perhaps the most immediately obvious effects of alcohol on the fetus is a pattern of abnormal facial features—small head circumference, skin folds at the corner of the eyes, small eye openings, low nasal bridge, short nose, small midface, thin upper lip, and flat philtrum^1^ (see figure, p. 33).

These facial abnormalities, however, are not present in all children who have been exposed to alcohol before birth. More subtle neuroanatomical and neurobehavioral problems often occur in alcohol-exposed children without facial abnormalities. Researchers and clinicians have used the term “fetal alcohol effects” (FAE) for many years to describe children exposed to alcohol before birth who do not have the FAS facial features but who exhibit other critical hallmarks of FAS. More recently, the Institute of Medicine (IOM) of the National Academy of Sciences has classified the effects of prenatal alcohol exposure into five categories (see table 1, p. 34) (Stratton et al. 1996)— three categories for children with all or some of the FAS facial features and two categories for children without any FAS facial features (i.e., ARBD and ARND). The diagnoses of both disorders require confirmation of the mother’s alcohol use during pregnancy in addition to a psychological or neurological assessment of the child. Without the identifiable FAS facial features, however, the categories ARBD and ARND are difficult to characterize.

## Effects of Prenatal Alcohol Exposure on Brain Structure and Function

Although not as obvious at birth as the characteristic facial features associated with FAS, the effects of alcohol-induced damage to the developing brain and spinal cord, or central nervous system (CNS), are just as deleterious. The problems that result from this damage become apparent during childhood and adolescence. They include reductions in general intellectual functioning and academic skills as well as deficits in verbal learning, spatial memory and reasoning, reaction time, balance, and other cognitive and motor skills. These deficits are particularly serious, because they are pervasive and persist throughout the person’s life. In fact, some deficits, such as problems with social functioning, appear to worsen as the sufferer reaches adolescence and adulthood, possibly leading to an increased rate of mental health disorders. A greater understanding of both the structural damage to the CNS from alcohol exposure (i.e., the “neuroanatomical” effects) and the resulting behavioral manifestations (i.e., the “neurobehavioral” effects) is critical to future research on effective therapies for FAS.

## Neuroimaging: Precise Pictures of Structural Damage to the Brain

Autopsies of the brains of children with FAS have demonstrated widespread and severe damage, including the following:

- Malformations of the brain tissue, both in the “gray matter” and “white matter” regions
- Failure of certain brain regions (e.g., the corpus callosum) to develop
- Failure of certain cells to migrate to their appropriate locations during embryonic brain development
- A tendency for the tissue to die in some brain regions (e.g., the cerebellum).

The extent of these abnormalities initially led researchers to conclude that neither a specific pattern of brain changes nor a consistent behavioral profile existed among children prenatally exposed to alcohol. However, the use of neuroimaging techniques, such as magnetic resonance imaging (MRI) and computed tomography, to visualize the living brain has provided a more precise picture of the brain structure of children with FAS, including reduced overall brain size. Several brain structures, such as the cerebellum and basal ganglia, appear to be especially sensitive to prenatal alcohol exposure. These findings suggest that alcohol exposure may cause specific, rather than global, developmental abnormalities.

### Cerebellum

The cerebellum appears to be especially affected by prenatal alcohol exposure. This structure is located at the back of the brain and is thought to be involved primarily in movement but also in cognitive processes, such as attention. Case studies and autopsy reports have shown that alcohol-exposed children with and without FAS exhibit smaller size and other abnormalities of the cerebellum. The death of certain cells in the cerebellum may be responsible for its reduced size. These specialized cells, called Purkinje cells, send out nerve signals in response to sensory and motor impulses from the rest of the nervous system.

### Basal Ganglia

The basal ganglia are paired masses of gray matter located deep within the white matter of the cerebrum. They include a structure called the caudate nucleus, which governs voluntary movement as well as some cognitive functions related to perception, thinking, and memory. The volume of the basal ganglia is significantly reduced in children with FAS compared with other children.

## Physical Measures of Altered Brain Function

In addition to pinpointing the structural changes in the brain, quantification of the resulting changes in brain function is of equal importance. If alcohol-induced changes in brain function could be identified early in a child’s life, scientists may possibly mitigate some of the adverse consequences as the child grows. Researchers have used two tools—acoustic analyses of babies’ cry patterns and electroencephalography (EEG)—to physically measure changes in brain function that may help diagnose and determine the prognosis even for alcohol-exposed children without full-blown FAS.

### Acoustic Cry Analyses

The characteristics of an infant’s cry, which are at least partly determined by the CNS, can be affected by prenatal exposure to alcohol and other drugs. In one study of 3-day-old infants, researchers examined three characteristics of crying of babies both with and without prenatal alcohol exposure: (1) the intensity at which a stimulus provokes crying (i.e., threshold); (2) the time between the stimulus and the infant’s cry (i.e., latency); and (3) the highness or lowness of the cry (i.e., pitch) (Zeskind et al. 1996). All three of these characteristics differed significantly between the two groups of infants. Moreover, the differences were related to the amount of alcohol consumed by the mother during her pregnancy. Although these findings indicate that prenatal alcohol exposure interferes with subtle aspects of neurobehavior, researchers have yet to determine how differences in cry characteristics may relate to later neurobehavioral outcomes, such as learning or attention.

### EEGs

EEG analyses and neurological testing also may help to identify less severe effects of alcohol exposure, such as ARND. Researchers have used EEG measurements to compare children with FAS, children with Down’s syndrome, and normally developing control subjects (Kaneko et al. 1996*a*,*b*). In those studies, children with prenatal alcohol exposure and children with Down’s syndrome had distinct profiles of brain electrical and neurological activity. The results suggested that the effects of prenatal alcohol exposure may specifically target the brain’s left hemisphere as well as differ from the effects of other congenital disorders, such as Down’s syndrome.

## Effects on Cognitive and Motor Functions

In recent years, researchers have investigated more specific aspects of brain functioning and behavior, rather than overall intellectual functioning, in exploring the effects of prenatal alcohol exposure. Two studies in both preschool (Janzen et al. 1995) and school-age (Mattson et al. 1998) children found that children with FAS, as well as alcohol-exposed children who did not meet all the FAS criteria, experienced deficits in numerous areas. The most prominent of these deficits were in integrating visual information with coordinated movements, controlling precise movements (e.g., speed and coordination of finger movements), language, and general intellectual functioning.

Although research has well established that heavy prenatal alcohol exposure leads to neurobehavioral impairment, the effects of lower levels of alcohol exposure are not as clear. Many of the problems linked to FAS also seem to exist in children whose mothers drank moderate amounts of alcohol when pregnant. These problems include deficits in general intellectual functioning, visual-spatial reasoning, attention, and academic achievement.

### Learning and Memory

To assess learning and memory in children with and without FAS, researchers gave the children a standardized test that showed differences in immediate recall, delayed recall, and recognition of words that had been read aloud (Mattson et al. 1996). The study found that the FAS children did not learn as many words as did the control group children, but the rate at which words were forgotten was the same in both groups. This finding suggests that children with FAS have profound deficits in learning when material is presented verbally but are capable of retaining the information they learn. Researchers have obtained similar results among prenatal alcohol-exposed children without a diagnosis of FAS.

Another study of implicit memory (i.e., the unconscious recall of a previously performed task) compared children with heavy prenatal alcohol exposure (including FAS), children with Down’s syndrome, and normally developing children (Mattson and Riley 1999). The study results suggested that the alcohol-exposed and the normally developing children were equally able to use previously learned information without being told to do so. In contrast, the children with Down’s syndrome were impaired on all memory tasks evaluated. Overall, these findings suggest that prenatal alcohol exposure does not impair some types of memory and that despite some learning deficits, children with FAS are able to retain learned information.

### Visual-Spatial Functioning

Only a few studies have assessed the visual-spatial functioning of persons with FAS—that is, the ability to see objects and understand their spatial relationships. Alcohol-exposed children appear to have deficits in specific aspects of visual-spatial processing, including perceiving and remembering spatial relationships and recalling visual details.

### Executive Functioning

Higher order cognitive processes called executive functions are activities that require complex thought processes and behaviors, such as planning, organizing, sequencing, and other forms of abstract thinking. Persons with deficits in these areas may have difficulty with self-care and independence. For example, routine activities that require a sequence of steps, such as getting dressed or writing a check, may be problematic.

Two studies evaluated the abilities of alcohol-exposed children in planning, verbal fluency, using information held in short-term memory, using feedback to modify behavior, and set shifting (e.g., switching from naming animals to naming furniture and back to animals) (Goodman et al. 1998; Kodituwakku et al. 1995). The alcohol-exposed children experienced more difficulties performing these tasks than did control children

In one of the studies, the alcohol-exposed children had difficulty only with certain tasks involving memory skills; in other areas, they tested similarly to the control group children (Kodituwakku et al. 1995). These findings support the conclusion that prenatal alcohol exposure targets specific areas of the brain.

In another study, teenagers and adults with FAS or FAE had more difficulty in calculating and estimating magnitude compared with control subjects (Kopera-Frye et al. 1996). Although the extent of these difficulties is unclear, they are related to lower overall intellectual ability. The study results support findings that persons with prenatal alcohol exposure experience specific problems in mathematics and problem-solving.

### Attention

Problems in maintaining attention have long been associated with FAS and are quite common, affecting 60 percent of children and adolescents with the syndrome. Deficits in attention also have been reported for children exposed to relatively low levels of alcohol before birth. Various studies indicate, however, that the attention disruption associated with prenatal alcohol exposure differs from that arising from other disorders, such as attention deficit hyper-activity disorder. Such differences could have important implications for diagnosing and treating attention disorders that are specifically attributable to prenatal alcohol exposure.

### Motor Control

Motor control is a complex function influenced by the CNS; by the peripheral nervous system, which provides feedback to the CNS from the body’s sensory organs (e.g., the eyes, ears, and skin); and by the vestibular system, which is located in the inner ear and is involved in a person’s sense of balance and the motor reactions used to maintain it. Defects in any of these systems can affect motor control.

Studies of humans and animals prenatally exposed to alcohol have consistently found impairments in the development of motor control. Further studies have suggested that balance deficits in alcohol-exposed persons may be attributable to problems of the CNS rather than the peripheral nervous system.

## Effects on Mental Health and Psychosocial Behavior

Both the psychosocial and psychiatric effects of prenatal alcohol exposure also profoundly influence the lives of alcohol-exposed children and their families. Impaired social functioning, disturbed behaviors, and psychiatric disorders are common in people with FAS. These problems, which can occur with or without mental retardation and persist into adulthood, often disrupt daily life and magnify other FAS-related problems.

### Mental Health

In a large study of secondary disabilities in persons of various ages with FAS or FAE, 94 percent of the participants had a history of mental health problems (Streissguth et al. 1996). Attention deficits were the most frequent problems in children and adolescents and occurred in 61 percent of the subjects. Among adults, depression was the most frequently reported problem (52 percent). Other studies found that preschool and school-aged children prenatally exposed to alcohol showed behaviors characteristic of people with autism, such as impairments in social interaction and communication.

### Psychosocial Behavior

Other studies have indicated additional impairments in social abilities and psychological functioning in alcohol-exposed children. For example, compared with control children, children prenatally exposed to alcohol had greater problems with respect to anxiety, social skills, and academic achievement; significantly higher scores on scales measuring behavioral problems, such as anxiety, depression, and attention problems; and more deficits in social skills, such as manners and interactions with others. The differences in social skills were greater at older ages, indicating that social skills developed more slowly in the FAS children.

## Underlying Mechanisms of Alcohol-Induced Damage to the Fetus

Current research on FAS seeks to delineate the specific mechanisms of damage to the fetus as well as the conditions that influence the extent of this damage. Numerous factors complicate this research. First, the process of development itself is enormously complex and not yet fully understood. Second, multiple distinct mechanisms work simultaneously along different biochemical pathways and at different physical sites in the developing embryo. And third, the ways in which these alcohol-induced mechanisms produce damage to the fetus depend on several variables, including the timing, frequency, and amount of maternal drinking during pregnancy; the mother’s health status and habits; and the genetic makeup of the mother and fetus.

Alcohol exerts its effects on the developing fetus through multiple actions at different sites. In the developing brain, for example, alcohol interferes with nerve cell (i.e., neuron) development and function in a variety of ways. Thus, in persons with FAS, certain brain regions have not developed normally, certain cells are not in their proper locations, and tissue has died off in some regions. At a different site in the developing embryo—the cell layer that develops into the bones and cartilage of the head and face—alcohol exposure at critical stages of development induces the premature cell death. This cell death is thought to be linked to the facial abnormalities found in FAS.

A developing embryo’s susceptibility to specific FAS defects appears to be directly related to the timing of maternal drinking, that is, whether drinking occurs during critical periods of vulnerability for different organ systems, regions, or cell types. Animal studies have also shown that the type and extent of fetal damage are related to the pattern of maternal drinking, with binge drinking being particularly damaging. Other factors modulating the effects of prenatal alcohol exposure include the particular profiles of blood alcohol concentrations produced, and the duration of exposure during development. Moreover, the effects of alcohol may be enhanced by other conditions that adversely affect the fetus, such as the use of tobacco and other drugs by the mother.

## Candidate Mechanisms for Central Nervous System Damage

Researchers have identified numerous mechanisms through which prenatal alcohol exposure may adversely affect brain development. These mechanisms act on a variety of brain cells and brain molecules. Although some of these mechanisms are specific to nervous system tissue, others also affect development of the craniofacial region or other body areas. Finally, regardless of the site of action, the timing, amount, and duration of alcohol exposure play a crucial role in determining the type and extent of damage.

### Timing of Exposure

In the developing brain, alcohol exposure during various stages of development can harm different populations of neurons through different processes. Some types of neurons are extremely vulnerable during the early stages of differentiation and when synapses are being formed. In other instances, neurons die when alcohol exposure either prevents them from migrating properly or induces a delayed cell death that occurs after migration, even though exposure occurred before migration started. Moreover, multiple mechanisms may operate simultaneously to produce abnormal cell development or cell death.

### Cell Death Modes

Cell death is the endpoint of many of the FAS mechanisms. It occurs by one of two recognized pathways: (1) necrosis, a reaction to injury or disrupted cell metabolism, or (2) apoptosis, a “programmed” self-destruction that is necessary for normal development but can be triggered to an excessive degree by toxins such as alcohol. While cell death by apoptosis is critical to healthy CNS development, this type of cell death also is involved in a broad range of human CNS disorders, including amyotrophic lateral sclerosis (ALS, or Lou Gehrig’s disease) and Alzheimer’s disease. Recent advances in knowledge about cell death modes provide the basis for FAS studies on the role of alcohol in inducing cell death in developing tissues.

### Free-Radical Damage

Free radicals are highly reactive molecular fragments that may be formed as a by-product of alcohol metabolism. Formation of these fragments likely plays an important role in producing cell damage in FAS, both in the CNS and in the craniofacial region. Numerous studies have indicated that alcohol may damage or kill fetal cells by causing the breakdown of mitochondria, a process that can be initiated by excessive amounts of free radicals. Antioxidants (e.g., vitamin C, vitamin E, and glutathione) are molecules that neutralize free radicals. The addition of antioxidants to cell cultures can prevent cell death, suggesting the potential for therapies with antioxidant treatment.

### Interference With Growth Factor Functions

Several chemicals, called growth factors, control cell proliferation and promote cell survival in the developing fetus. Current research indicates that alcohol exposure may disrupt the developing CNS by interfering with the production or function of some of these growth factors. These include molecules called insulin-like growth factors, nerve growth factor, basic fibroblast growth factor, and a neurotrophic growth factor.

### Adverse Effects on Astrocyte Formation

Astrocytes are star-shaped cells of the nervous system that, unlike neurons, do not actively transmit information to other cells. Nevertheless, astrocytes interact intimately with neurons and other astrocytes and play critical roles in the developing CNS. For example, cells that are precursors to astrocytes guide migrating neurons to their appropriate destinations in the brain. One possible mechanism for alcohol-induced abnormalities in the fetus involves errors in the process of astrocyte formation. Such alcohol-induced defects in astrocyte formation could explain why certain neurons are not found in the appropriate places in the brain after the normal migration period. Recently, researchers also found that alcohol interferes with the normal growth and function of astrocytes.

### Abnormal Development of Neurotransmitter Systems

Neurotransmitters are chemicals that allow communication among nerve cells. The neurotransmitters are released from an extension (i.e., the axon terminal) of the signal-emitting neuron, travel across a narrow gap between neurons, and bind to specific docking molecules (i.e., receptors) on the target neuron. Alcohol significantly interferes with two neurotransmitter systems that play important roles in fetal brain development, the serotonin system and the glutamate system. In rats, early prenatal alcohol exposure significantly delays the development of serotonin-using neurons, reducing serotonin levels and altering the binding of serotonin to receptors in many target sites during periods that are likely to be critical for normal brain development. Similarly, prenatal alcohol exposure, even in moderate concentrations, results in a decrease in the number and function of certain glutamate receptors—called *N*-methyl-d-aspartate (NMDA) receptors—throughout development. Alcohol’s effects on NMDA receptors during critical periods of brain development may play a major role in the mental and behavioral deficiencies found in FAS.

### Altered Glucose Transport and Uptake

All cells need the sugar glucose in order to generate energy, metabolize free radicals, and synthesize vital chemicals, including neurotransmitters and nucleic acids. Several studies have demonstrated that alcohol can impair glucose transport and uptake during development. For example, cell culture studies show that alcohol exposure can reduce glucose uptake by certain brain neurons from fetal rats and by astrocytes from newborn rats. Further research on the mechanisms of glucose transport and uptake could contribute significantly to knowledge about alcohol’s effects on developing cells.

### Abnormal Cell Adhesion Molecules

Cell adhesion molecules influence the ability of CNS cells to migrate properly; to develop branching extensions, such as axons and dendrites; and to survive. Defects in one particular cell adhesion molecule, called L1, can lead to abnormalities in brain development and mental deficiencies that are similar to those seen in children with FAS. Some cell culture studies have shown that low levels of alcohol interfere with the ability of L1 to regulate the clustering or clumping together of cells that is needed for brain structures to develop.

### Altered Regulation of Gene Expression

The process of converting a gene’s encoded information into a gene product (e.g., a protein) is called gene expression. In alcohol research, scientists are particularly interested in the expression of certain genes (i.e., homeobox genes) that regulate the activation and timing of steps in the formation of specialized tissues and organs in the body. Although researchers know that alcohol can affect the expression of some genes, it is uncertain whether these include homeobox genes. The lack of information on how alcohol affects the regulation of genes that control the formation of the CNS and other body parts creates a major gap in the current understanding of the mechanisms underlying FAS. Consequently, studies on alcohol-induced changes in gene expression during critical periods of development constitute one of the most promising areas for new FAS research.

## Candidate Mechanisms for Craniofacial Defects

In mouse and chicken embryos, as in humans, heavy exposure to alcohol during certain periods of development can give rise to the craniofacial abnormalities associated with FAS. Animal studies have linked these abnormalities to cell death by apoptosis of certain embryonic cells, called neural crest cells, during a very defined and narrow period of vulnerability in early embryonic development. One mechanism by which this occurs is thought to be the formation of free radicals. Two other possible mechanisms are a deficiency in retinoic acid and altered expression of homeobox genes.

Retinoic acid is a derivative of vitamin A (retinol), which is essential for controlling the normal pattern of development of tissues and organs in vertebrate animals, including the development of neural crest cells into craniofacial features. Retinoic acid likely acts in this capacity by binding to receptors that regulate the expression of homeobox genes. Thus, certain retinoic acid receptors control the specific homeobox genes that regulate the timing and coordination of craniofacial development. Alcohol exposure at specific periods of embryonic development can reduce the production of retinoic acid. Deficiencies or abnormalities in retinoic acid or its receptors, in turn, cause neural crest cells to die by apoptosis, leading to craniofacial defects.

## Issues in FAS Prevention

Unlike most other birth defects, FAS has the potential to be entirely preventable, because its direct cause—maternal drinking—is presumed to be a controllable behavior. Although many strategies to prevent FAS have been developed and implemented in recent years, an intensifying need exists for effective prevention strategies. One study found that although alcohol use among pregnant women decreased between 1988 and 1992 (from 22.5 to 9.5 percent), by 1995 it had increased to 15.3 percent (Ebrahim et al. 1998). Moreover, binge drinking (defined in the study as five or more drinks per occasion) among pregnant women, a particularly hazardous drinking pattern in terms of FAS risk, increased significantly between 1991 and 1995 (from 0.7 to 2.9 percent of pregnant women) (Ebrahim et al. 1999). In addition, little is known about the patterns of drinking by pregnant women; the social and psychological risk factors associated with drinking during pregnancy and the birth of FAS children; or the processes by which drinking, particularly heavy drinking, by pregnant women can be prevented. Consequently, the generation of a solid research base to guide prevention program developers is critical.

## Reviews of Prevention Programs and Research

Several investigators have reviewed the literature on FAS prevention in order to identify effective approaches as well as shortcomings of the current literature. One review of more than 160 articles described treatment programs designed to reduce fetal alcohol exposure and damage in alcohol- and drug-dependent women (Finkelstein 1993). This analysis concluded that programs that provide comprehensive and coordinated treatment could best draw pregnant women into care and provide the most effective treatment for alcohol-abusing women. Such comprehensive approaches include social, cognitive-behavioral, medical, and referral services that should be coordinated through an active case manager for greatest effectiveness. In addition, other reports have indicated that comprehensive programs should include active outreach strategies to attract high-risk drinkers and should offer family support and counseling services.

Another review (May 1995) emphasized a public health approach to the prevention of FAS. This approach classifies prevention strategies into the three levels of prevention commonly used in the field of public health:

- Primary prevention approaches that attempt to stop maternal drinking before it starts
- Secondary approaches that facilitate early detection and treatment of maternal drinking problems before they lead to FAS
- Tertiary approaches that attempt to change the behaviors of women who are at very high risk because they have already delivered a child with diagnosable FAS or other alcohol-related disorders, such as ARBD and ARND.

More recently, the IOM’s Committee to Study Fetal Alcohol Syndrome reviewed a vast body of FAS literature and proposed its own comprehensive recommendations that include a variety of prevention measures (Stratton et al. 1996). These measures fall into three categories as follows:

- Universal approaches attempt to promote the health and well-being of all people in society or in a particular community, without regard to individual risk, through use of the media to educate the public and through policy and environmental change.
- Selective preventive interventions target persons and subgroups that are at excess risk of developing the problem, such as women of child-bearing age who drink alcohol. These interventions should be given by health care providers who are trained to question women about their drinking and contraceptive histories and to deliver interventions that are proportional to the woman’s level of risk.
- Indicated interventions are targeted to women who are at high risk of giving birth to an alcohol-impaired child, because, for instance, they are drinking at a level that is likely to produce FAS-affected offspring or they have already delivered one child with FAS. These interventions should be offered in the form of brief interventions or more formal approaches as needed.

### Universal Prevention Approaches

Most women reduce or cease their drinking during pregnancy. This reduction may be linked, among others, to universal prevention messages in reading material and in radio and television advertisements. One universal prevention strategy is the use of alcoholic beverage labels that warn about the risks of birth defects if women drink alcohol during pregnancy. However, warning labels appear to have a preventive effect on lighter drinkers but not on women who are the heaviest drinkers and who are thereby at greatest risk of bearing a child with FAS. Women who are the heaviest and most long-term drinkers also tend to show the least amount of change in their drinking behavior once they become pregnant.

### Selective Prevention Approaches

Much information regarding risk factors for FAS (e.g., age, socioeconomic status, and spousal characteristics) is available and can help provide appropriate population targets for selective and indicated prevention strategies. The first challenge in implementing such prevention strategies is to identify accurately and efficiently women at increased risk of having FAS children through screening in settings such as clinics that provide primary and prenatal care to low-income women. Some guidelines are available for detecting higher risk drinkers in primary care settings.

The identification of problem drinkers primarily relies on the use of short screening questionnaires. Many of the most commonly used questionnaires, however, are less accurate for women than for men. Reasons for this difference probably include the increased stigma experienced by women who drink, which may lead them to under-report alcohol problems. Women are also less likely to experience some of the more adverse consequences of drinking, such as employment, economic, or social problems; thus, standard screening instruments may fail to identify women whose drinking problems are expressed in other ways.

Attempts to improve identification of women who drink during pregnancy have focused on comparing the accuracy of various screening instruments with each other and with informal questioning by health care workers. In one study (Chang et al. 1998), the T-ACE questionnaire (see table 2) was more effective than assessments by health care staff in identifying pregnant women at risk for problem drinking. More work is needed to develop instruments for use among general populations of women of childbearing age.

Once women at risk for having children with FAS are identified, several other questions become paramount, such as the women’s readiness for change, the factors that affect readiness, and actual turning points toward abstinence. These issues must be addressed and evaluated as they are in other alcohol research that does not involve pregnant women.

### Indicated Prevention Approaches

Effective approaches to FAS prevention among the highest risk women, particularly mothers who have previously given birth to an alcohol-impaired child, would eliminate most of the existing FAS problem, because these women account for the majority of FAS cases. Reaching these high-risk women is problematic, however. For example, many heavily drinking pregnant women never present themselves to prenatal clinics and are otherwise elusive. If they receive therapy for their alcohol dependence, such treatment rarely includes an emphasis on FAS prevention. Furthermore, barriers to treatment clearly exist for these highest risk women.

For those women identified as having the greatest risk of an FAS birth, a wide range of indicated prevention strategies exists, and researchers must determine which types of therapy are most effective for which subtypes of women. Specifically, investigators should compare the effectiveness of brief versus extended interventions; coercive versus voluntary therapies, such as motivational enhancement; and group versus individual approaches. Furthermore, studies should evaluate various prototypes of case management, social network therapy, support groups for FAS mothers, environmental change, and “social model” approaches to recovery, such as the community reinforcement approach. Currently used indicated prevention strategies include the following:

- Intensive case management for alcohol-abusing women who have had FAS children
- Programs that combine alcohol interventions with the promotion of contraceptive use
- “Aftercare” programs for women who have given birth to children with FAS.

However, researchers have not yet determined the effectiveness of some of these approaches.

The IOM report (Stratton et al. 1996) recommends that any health care provider who encounters a woman who is abusing alcohol should consider brief intervention therapy, counseling regarding the risks of prenatal alcohol exposure, and (if appropriate) referral to more formal alcohol abuse treatment. For women who continue to abuse alcohol during pregnancy, comprehensive clinical treatment programs may be necessary. These programs generally include medical and obstetric care in addition to alcohol and other drug abuse services, which in turn involve individual or group counseling, family therapy, referral to self-help groups, parenting skills training, and case management, as well as information on the effects and risks of alcohol consumption.

Some communities have mandated court-ordered or involuntary participation in alcohol abuse treatment for heavily drinking pregnant women as a means of preventing FAS. These types of coercive programs have stimulated legal and ethical debates in the literature concerning the comparative rights of the pregnant woman, the fetus, and society at large. Furthermore, the effectiveness of this approach has not been determined, although some encouraging findings exist. Consequently, evaluations comparing treatment completion rates and changes in actual drinking behavior of women for whom treatment had been mandated and heavily drinking pregnant women without mandated treatment are needed.

## Summary

Imaging studies have demonstrated abnormalities of certain brain regions in persons exposed to alcohol prenatally, whereas other regions seem to be spared structural damage. Similarly, research shows that many neurobehavioral deficits are notably linked to prenatal alcohol exposure, whereas other functions appear to remain intact. These studies strongly support the notion that alcohol has specific, rather than global, effects on the developing brain.

Advancements in our understanding of mechanisms of FAS damage will guide the development of new ways to protect against or limit alcohol-induced damage to the fetus. For example, the identification of specific mechanisms and biochemical markers of damage should accelerate early detection or allow better prediction of specific types of damage in at-risk pregnancies. Such advances could help identify cases at greatest risk for developmental disorders and improve outcomes through targeted interventions. From a public health perspective, knowledge of specific mechanisms of damage should be a powerful tool for effective public education and counseling of alcohol-dependent women in their childbearing years and could help guide clinical decisions about the most effective allocation of medical and psychological support services.

Progress in the prevention of FAS must begin with research that establishes baseline information about the prevalence of FAS and identifies more precisely those women who are at highest risk of bearing an alcohol-affected child. Equally important, the effectiveness of different FAS prevention approaches must be determined through carefully controlled evaluation studies. Each of the levels of prevention (i.e., universal, selected, and indicated prevention), as well as the specific modalities used within each, must be examined both in isolation and as part of comprehensive programs. Because FAS and other adverse effects of drinking during pregnancy theoretically are 100-percent preventable, it is vital to make every effort to achieve this goal.

## Figures and Tables

**Figure 1:**
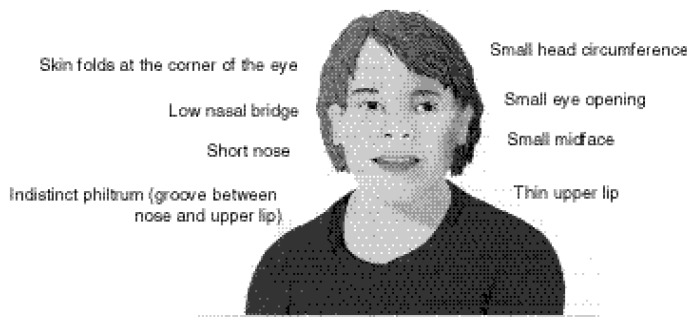
Facial features of FAS

**Table 1:** Criteria for Diagnosing the Effects of Prenatal Alcohol Exposure

Diagnosis	FAS Facial Features	Confirmed Prenatal Alcohol Exposure	Additional Criteria
Fetal Alcohol Syndrome (FAS) with confirmed maternal alcohol exposure	Yes	Yes	
FAS without confirmed maternal alcohol exposure	Yes	No	Growth retardation; central nervous system (CNS) abnormality; or evidence of a behavioral or cognitive disorder inconsistent with the expected developmental level, with hereditary factors, or with the environment
Partial FAS with confirmed maternal alcohol exposure	Some	Yes	
Alcohol-related birth defects (ARBD)	No	Yes	Any of a number of anomalies (e.g., heart or kidney defects) present at birth that are associated with maternal alcohol consumption during pregnancy
Alcohol-related neurodevelopmental disorder (ARND)	No	Yes	Evidence of CNS abnormality (e.g., abnormally small head, abnormal brain structures, and specific neurological signs); evidence of a behavioral or cognitive disorder inconsistent with the expected developmental level, with hereditary factors, or with the environment; or evidence of both

SOURCE: Stratton et al. 1996. Reprinted with permission from *Fetal Alcohol Syndrome: Diagnosis, Epidemiology, Prevention, and Treatment.* Copyright 1996, National Academy of Sciences, Washington, DC.

**Table 2:** Commonly Used Screening Questionnaires for Identifying Problem Drinking

**CAGE** Have you ever felt you should Cut down on your drinking? Have people Annoyed you by criticizing your drinking? Have you ever felt bad or Guilty about your drinking? Have you ever had a drink first thing in the morning to steady your nerves or get rid of a hangover (Eye opener)? Each item receives a score of 1 for a positive response (Ewing 1984).
**T-ACE** Tolerance—How many drinks can you hold? Have people Annoyed you by complaining about your drinking? Have you ever felt you ought to Cut down on your drinking? Eye opener—Have you ever had a drink first thing in the morning to steady your nerves or get rid of a hangover? A score of 2 is given for a positive response to the tolerance question; 1 point each is scored for the other three questions (Sokol 1989).
**TWEAK** How many drinks can you hold? (Tolerance) Does your spouse [or do your parents] ever Worry or complain about your drinking? Have you ever had a drink first thing in the morning to steady your nerves or get rid of a hangover? (Eye opener) Have you ever awakened in the morning after drinking the night before and found that you could not remember a part of the evening before? (Amnesia) Have you ever felt you ought to cut [Kut] down on your drinking? Positive answers to the tolerance and worry questions score 2 points each; the other three questions score 1 point each (Chan et al. 1993).
**MAST (Michigan Alcoholism Screening Test)** Consists of 25 questions, each weighted 0, 1, 2, or 5, and when summed yielding scores of 0 to 53 (Selzer 1971).

## Footnotes

1 For a definition of this and other technical terms used in this article, see the glossary, p. 41.

## GLOSSARY

Antioxidant: Substances that inactivate, or neutralize, *free radicals*.
Autism: A developmental disorder characterized by severe social, communication, and behavioral problems.
Axon: Extension of a *neuron* that transmits nerve signals to other *neurons*.
Basal ganglia: A group of structures deep inside the brain that are involved in movement and cognition.
Cerebellum: A region at the base of the brain that primarily is involved in the coordination of movement.
Cerebrum: The largest portion of the brain; involved in controlling consciousness, voluntary processes, and cognition.
Corpus callosum: The central tract inside the brain that connects the right and left halves, or hemispheres, of the brain.
Delayed recall: Ability to recall information (e.g., certain words) after a short interval (e.g., 20 minutes).
Dendrite: Extension of a *neuron* that receives nerve signals from other neurons.
Free radical: Highly reactive molecular fragments; can be formed during alcohol metabolism.
Gray matter: Brain tissue that consists mostly of nerve cell bodies and *dendrites*.
Immediate recall: Ability to recall information (e.g., certain words) immediately after hearing them.
Mitochondria: Small structure inside a cell where the cell generates most of its energy.
Neuron: Nerve cell.
Nucleic acid: Molecules that make up the cell’s genetic material (e.g., the DNA).
Philtrum: The groove between the nose and the upper lip.
Synapse: The point of contact between two *neurons* at which nerve signals are transmitted from one cell to the other.
White matter: Brain tissue that consists mostly of *axons*.
